# Development and Clinical Validation of Novel 8-Gene Prognostic Signature Associated With the Proportion of Regulatory T Cells by Weighted Gene Co-Expression Network Analysis in Uterine Corpus Endometrial Carcinoma

**DOI:** 10.3389/fimmu.2021.788431

**Published:** 2021-12-14

**Authors:** Jinhui Liu, Rui Geng, Sheng Yang, Fang Shao, Zihang Zhong, Min Yang, Senmiao Ni, Lixin Cai, Jianling Bai

**Affiliations:** ^1^ Department of Gynecology, The First Affiliated Hospital of Nanjing Medical University, Nanjing, China; ^2^ Department of Biostatistics, School of Public Heath, Nanjing Medical University, Nanjing, China

**Keywords:** uterine corpus endometrial carcinoma, regulatory T cells, immunotherapy, prognostic markers, TCGA

## Abstract

**Background:**

Uterine corpus endometrial carcinoma (UCEC) is a gynecological malignant tumor with low survival rate and poor prognosis. The traditional clinicopathological staging is insufficient to estimate the prognosis of UCEC. It is necessary to select a more effective prognostic signature of UCEC to predict the prognosis and immunotherapy effect of UCEC.

**Methods:**

CIBERSORT and weighted correlation network analysis (WGCNA) algorithms were combined to screen modules related to regulatory T (Treg) cells. Subsequently, univariate, least absolute shrinkage and selection operator (LASSO), and multivariate Cox regression analyses were used to identify the genes in key modules. The difference in overall survival (OS) between high- and low-risk patients was analyzed by Kaplan–Meier analysis. The Tregs-related risk signature (TRRS) was screened by uni- and multivariate Cox analyses. Afterward, we analyzed the expression difference of TRRS and verified its ability to predict the prognosis of UCEC and the effect of immunotherapy.

**Results:**

Red module has the highest correlation with Tregs among all clustered modules. Pathways enrichment indicated that the related processes of UCEC were primarily associated to the immune system. Eight genes (ZSWIM1, NPRL3, GOLGA7, ST6GALNAC4, CDC16, ITPK1, PCSK4, and CORO1B) were selected to construct TRRS. We found that this TRRS is a significantly independent prognostic factor of UCEC. Low-risk patients have higher overall survival than high-risk patients. The immune status of different groups was different, and tumor-related pathways were enriched in patients with higher risk score. Low-risk patients are more likely take higher tumor mutation burden (TMB). Meanwhile, they are more sensitive to chemotherapy than patients with high-risk score, which indicated a superior prognosis. Immune checkpoints such as PD-1, CTLA4, PD-L1, and PD-L2 all had a higher expression level in low-risk group. TRRS expression really has a relevance with the sensitivity of UCEC patients to chemotherapeutic drugs.

**Conclusion:**

We developed and validated a TRRS to estimate the prognosis and reflect the immune status of UCEC, which could accurately assess the prognosis of patients with UCEC and supply personalized treatments for them.

## Introduction

Uterine corpus endometrial carcinoma (UCEC) is the third most common gynecological malignant cancer globally (1). In 2019, the incidence and mortality of UCEC have been estimated at 61,880 and 12,160, respectively, merely in the United States (2). Obesity can increase the risk of uterine corpus endometrial carcinoma (3). At present, multiple treatment such as surgery, chemotherapy, radiation therapy, and hormone therapy are always applied to UCEC treatment, but the incidence and disease-related mortality are still increasing annually (4–6). Effective treatment is based on accurate assessment of prognosis. Nevertheless, patients in the same clinical stage may present different clinical characteristics, indicating that the prognosis of UCEC according to the traditional clinicopathological staging is not fully accurate (7). Consequently, using effective biomarkers to accurately define different UCEC stage is helpful to precise treatment. In recent years, immunotherapy has become an effective therapy for cancer (8), especially in melanoma, lung cancer, and liver cancer (9). Tregs, a category of CD4+ T cells, can maintain immune homeostasis through regulating antimicrobial resistance, allergy, and transplantation rejection, and suppressing protective immune responses (10). It has been confirmed that Tregs could be employed to predict the outcomes of solid tumors like breast and ovarian (4, 11). Checkpoint activity of T cells can be suppressed by immune checkpoint inhibitor (ICI). In particularly, CTLA‐4 and PD‐1 are important ICI (12, 13) and have shown good efficacy in cancer treatment (4). More recently, olaparib has shown a clinical effect on UCEC to certain degree (14); the relationships between olaparib exposure and UCEC biomarkers are still unknown (1). The expression of RNF183, which has been considered as a good prognostic marker of UCEC, has been confirmed to be related to the markers of different subsets of Tregs (15). Therefore, the identification of TRRS will be helpful to explore the immunotherapy of UCEC.

With the continuous development of bioinformation technology, a great deal of methods has been employed to define biomarkers. WGCNA algorithm can identify highly correlated modules and genes for cancer based on the network construct of genes expression (16). This study applied WGCNA to identify relevant modules and genes of UCEC, and the red module was selected for further analysis. In addition, we construct a prognostic model related to Tregs and analyzed its relationship with immune microenvironment, chemotherapy, and immunotherapy. Furthermore, the TRRS might be used as a novel tool to diagnose UCEC patients and provide more effective personalized treatment.

## Materials and Methods

### Data Acquisition

A total of 552 UCEC cases and 23 normal samples were obtained from The Cancer Genome Atlas (TCGA) data portal (https://portal.gdc.cancer.gov/) (17). Clinical factors data such as age, grade, and histological type were downloaded from TCGA data portal.

### Estimation Tregs Proportion and Differential Analysis in UCEC

CIBERSORT was employed to estimate the proportion of 22 immune cells for normal and tumor tissues (18). Therefore, we identified 309 samples with the standards of p < 0.05 from 575 cases of samples.

### Construction of Co-expression Network and Select Hub Module

*Intersecting Gene and Low-Tregs Groups.* Second, we used Limma to define the differentially expressed genes with a p-value lower than 0.05 and log foldchange larger than 1 as the threshold (19). We obtained 4,703 genes. Then, these genes were used to construct a WGCNA (20). First, according to the Pearson’s correlation value, a weighted matrix was constructed. Next, amn = |cmn| β (cmn means Pearson’s correlation value of paired genes; amn is adjacency between paired genes) was used to construct a weighted adjacency matrix. Parameter β is a soft threshold, has the function of strengthening correlations, and reduce weakening correlations between genes. The value of β was defined as 6. For the purpose of dividing the genes with resembling expression levels into different modules. We cluster these genes with minimum size genes dendrogram of 50. The key module was identified through the analysis of the correlation between these genes and clinical factors. Eigengene dendrogram and adjacency heatmap also confirmed that the red module has a higher correlation coefficient. A protein–protein interaction (PPI) network was constructed base on STRING database, a tool that integrates all the connections between proteins of interacting genes (21), intuitively showing the interacting nodes of each genes in the red module.

### Gene Set Enrichment Analysis

Enrichr (https://maayanlab.cloud/Enrichr/) is a web-based program that has plenty information about protein and gene, in which investigators always use to do pathway and process enrichment analysis (22). For further analysis, Enrichr was applied to conduct Gene Ontology (GO) and Kyoto Encyclopedia of Genes and Genomes (KEGG) to analyze the functions of genes in the red module.

### Construction of TRRS

We obtained 537 samples with complete gene expression profiles and OS time. We randomly assigned 269 patients as the train set based on a computer-generated allocation sequence and took the entire set as the validation data. The train set was employed to construct TRRS, while the entire set was chosen for validating the predicting value. LASSO analysis based on R package “glmnet” combined with multivariate Cox regression analyses had been employed to select genes that has significant connections with OS of UCEC. Afterwards, these genes were applied to construct TRRS (23). The coefficients calculated by LASSO regression were applied to obtain the formula as follows: risk score = sum of coefficients × 8 TRRS expression level (24). All the samples were split into two groups in the light of the median risk score. Through Kaplan–Meier curves, receiver operating characteristic curves (ROC), and principal component analysis, we assess the accuracy of TRRS. Risk score distribution, survival status, and genes expression were also taken into consideration. In addition, univariate and multivariate Cox regression analyses were applied to calculate the predictive ability of TRRS.

### Quantitative Real-Time RT-PCR

Total RNA from 16 UCEC samples and 16 normal tissues was extracted on the basis of Trizol reagent (Invitrogen). Before reverse transcription to cDNA, 4× GDNA wiper mix (vazymer323-01) was employed to remove residual genomic DNA from total RNA. Complementary RNA was synthesized by using PrimeScript RT reagent kit. Real-time quantification was performed using the SYBR Premix Ex Taq Kit (TaKaRa DRR041). The relative expression level of the target gene was standardized by GAPDH and 2^−△△Ct^ method. Quantitative real-time RT-PCR (qRT-PCR) primers are listed in **Supplementary Table S1**.

### Construction of a Nomogram

Five clinical characteristics such as age, stage, grade, histological type, and risk score were combined to establish a nomogram aims to calculate the OS of 1, 3, and 5 years of UCEC in the entire set. Calibration curves were employed to evaluate whether the established nomogram is reliable (25).

### Immune and Clinical Characteristic Identification

A total of 552 UCEC samples and 23 normal samples were obtained from TCGA database, which was employed to verify the expression distinction between tumor and normal tissues. Furthermore, boxplots were drawn to analyze the relationship between gene expression and clinical factors. Finally, according to the level of expression, patients were split into two groups. Then, we verified the connection between TRRS expression and survival probability based on K–M analysis.

### Genome-Wide Analysis of Genes

Gene Set Cancer Analysis (GSCALite), a web server, aims to flexibly calculate the expression, mutation, and interaction of genes in different cancers (26). GSCALite has the function to analyze different gene expression levels, survival time, mutations, methylation, chemotherapeutics sensitivity, and so on. We analyze mutation distribution and global activity of eight genes. After that, we identified the connection between genes expression levels and copy number variations (CNVs) and methylation by using GSCALite (27).

### Survival Analyses of Genes in TRRS

The areas under the curve (AUCs) of 1, 3, and 5 years on account of risk score and different clinical factors were calculated, respectively. Furthermore, the curve was employed to identify the total influence of risk score combined with clinical characteristics on survival probability. Overall survival rates of different clinical characteristics were calculated as well.

### IPS Analysis

Immunomodulators, major histocompatibility complex (MHC) molecules, effector cells, and immunosuppressive cells are the four main components to evaluate tumor immunogenicity. Through calculating the expression values of four kinds of immune genes, we derived a patient’s immunophenoscore (IPS). The IPS was achieved based on a scale range from 0 to 10. The higher the score, the stronger the immunogenicity. The IPSs (including IPS, IPS-CTLA4, IPS-CTLA4/PD-L1/PD1/PD-L2, and IPS-PD1/PD-L1/PD-L2 scores), acquired from The Cancer Immunome Atlas [TCIA (https://tcia.at/home)] (28), were applied to assess the response of UCEC patients for ICI.

### Estimation of ICIs Response

The connection between ICI expression and risk score of UCEC patients was identified based on Pearson correlation coefficient method. Boxplots display the results.

### GSEA and ESTIMATE and ssGSEA

Function and pathways for different risk groups were investigated by GSEA, respectively (29). Pathways with nominal *p* < 0.05 were considered significantly enriched. Immune scores, stromal scores, and estimate scores of each sample were received based on the “Estimation of Stromal and Immune cells in Malignant Tumors using Expression data” (ESTIMATE) algorithm (30). Additionally, the difference in immune cell and immune function between two risk groups were achieved by CIBERSORT method. Single-sample gene set enrichment (ssGSEA) is another method to identify the distinction of immunological status between patients in different risks. ssGSEA utilized the enrichment scores to indicate the degree of absolute enrichment in each sample. Standardized enrichment scores for each immune category could be calculated (31).

### Mutation Analysis

The mutation materials of UCEC were obtained from TCGA. Somatic mutation data are stored in mutation annotation format (MAF) (32). The TMB scores of every patient were calculated based on the following formula: TMB = (total mutation/total covered bases) × 10^6^.

### Chemotherapeutic Response Prediction

A total of 537 samples were split into high- and low-risk groups on the basis of median risk score, and each group was given six kinds of chemotherapeutics including cisplatin, docetaxel, doxorubicin, gemcitabine, methotrexate, and paclitaxel. The sensitivity of each sample to chemotherapy was predicted by using Genomics of Drug Sensitivity in Cancer (GDSC) (https://www.cancerrxgene.org/) (33). The calculation was conducted through R package “pRRophetic”, where the samples’ half-maximal inhibitory concentration (IC50) was achieved by ridge regression. Pearson correlation coefficient was applied to explore the relevance between gene expression in TRRS and their sensitivity to pharmacotherapy.

### Immunoassay for ZSWIM1

For the purpose of further studying the correlations between ZSWIM1 and immunity, tumor–immune system interactions and drugbank (TISIDB, http://cis.hku.hk/TISIDB/index.php) database were applied and inferred the correlation between ZSWIM1 expression and the TRRS of UCEC (34).

### Statistical Analysis

R (version 4.0.5) was applied to perform statistical analysis in our study. All statistical tests were two-sided, and *p* < 0.05 was considered as statistical difference. Student’s t-test was used to make a comparison between the normally distributed variables in the two groups, and Wilcoxon test was used to calculate the continuous variables.

## Results

### Tregs in Uterine Corpus Endometrial Carcinoma

**Figure 1:**
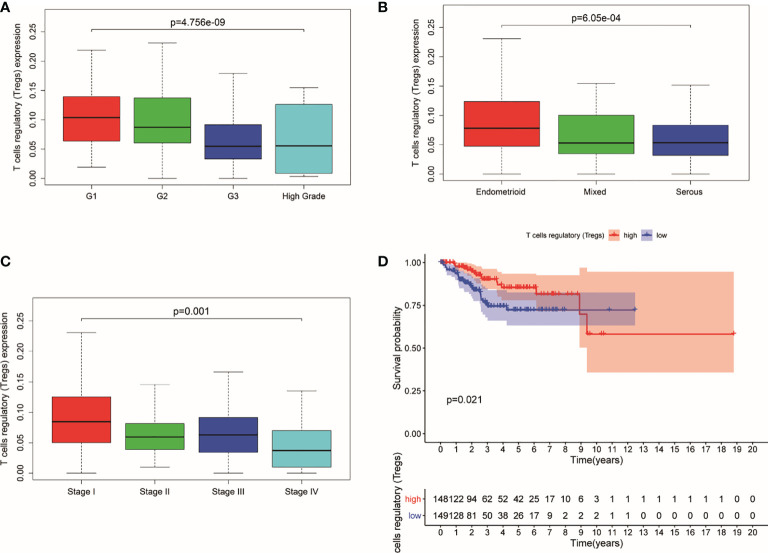
Expression of Tregs in different stages and its relationship with survival probability. **(A)** Expression of Tregs in different pathological grades. **(B)** Expression of Tregs in different pathological morphology. **(C)** Expression of Tregs in different pathological EC stages. **(D)** Survival probability between patients with high and low T cells regulatory.

The study indicated that a lower expression of Tregs was related to higher clinical grade and more serious pathological morphology (*p* = 4.756e−09, *p* = 6.05e−04, respectively) (**Figures 1A, B**). Meanwhile, patients in advanced stage had a lower expression of Tregs in general (*p* = 0.001) (**Figure 1C**). Additionally, the prognosis of UCEC patients with lower Tregs expression is poorer than those with higher Tregs expression (**Figure 1D**).

### Construct Weighted Co-Expression Network

**Figure 2:**
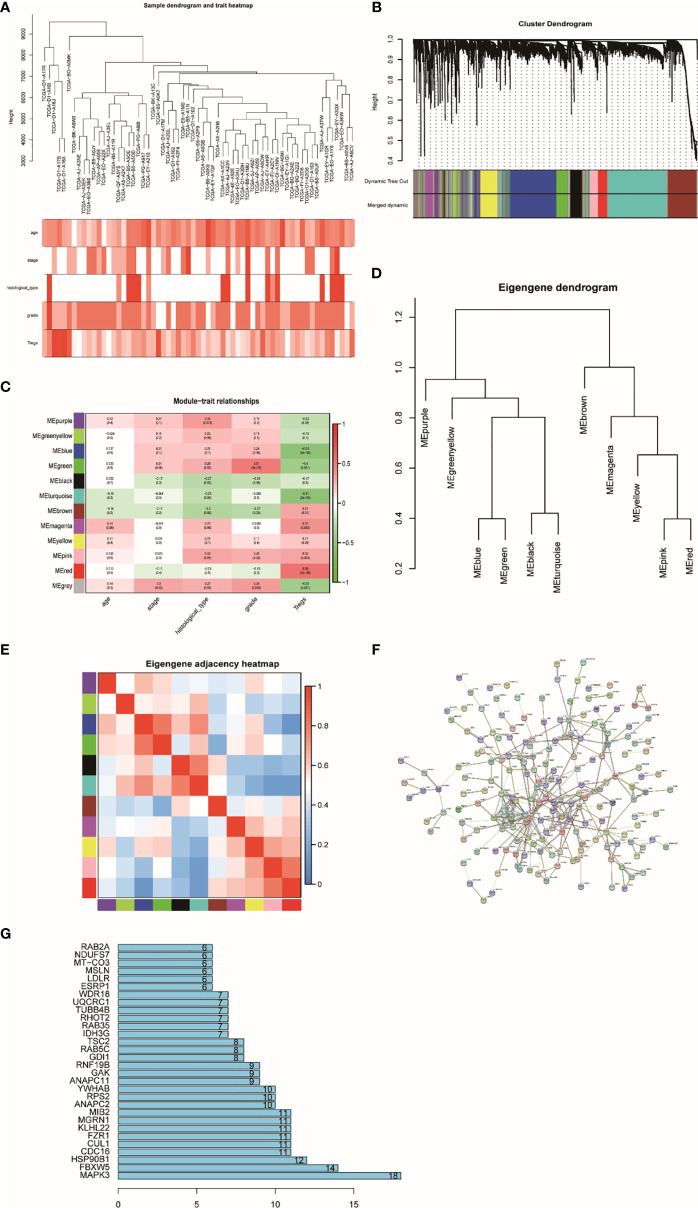
Sample clustering and correlation analysis. **(A)** Sample dendrogram and trait indicator: in the heat map, the darker the color, the stronger the correlation between samples and clinical traits. **(B)** Clustering dendrogram of 4,703 genes with difference in Tregs and 12 gene modules from 298 UCEC. **(C)** Heatmap of the correlation between module eigengenes and clinical characteristics of UCEC. **(D)** Randomly selected partial genes to make network heatmap plot. **(E)** Eigengene dendrogram and adjacent heatmap. **(F)** PPI network constructed using STRING. **(G)** Bar graph of the top 30 genes that have more connections with other genes.

A total of 4,703 genes were applied to set up a weighted co-expression network based on WGCNA. First, combining with clinical factors, we clustered 298 samples and constructed samples dendrogram and trait heatmap for them (**Figure 2A**). Then, we identified the value of β = 6 (R^2^ = 0.91) as the threshold (**Supplementary Figures S1A, B**). **Supplementary Figures S1C, D** indicate positive result of rationality test. In addition, a hierarchical clustering tree had been established, and 12 modules were generated (**Figure 2B**). Genes having relative expression were clustered together to form a branch that constructed a module. Among the 12 modules formed by clustering, the correlation between the red module and Tregs is higher than that between other modules, which have 251 genes (**Figure 2C**). **Figures 2D, E** show that the modules are not independent of each other. Finally, PPI network was constructed by using STRING (**Figure 2F**), which indicates that most genes are connected with the others in the red module. **Figure 2G** shows the top 30 genes that have more connections with other genes.

### Pathway and Process Enrichment Analysis

We employed GO and KEGG analysis to conduct enrichment analysis and find out the functions and pathways that related to the red module. The outcome indicated that the functions and pathways of UCEC were primarily bound with immune-related physiological processes (**Supplementary Figure S2**).

### Key Genes Identification and TRRS Construction

**Figure 3:**
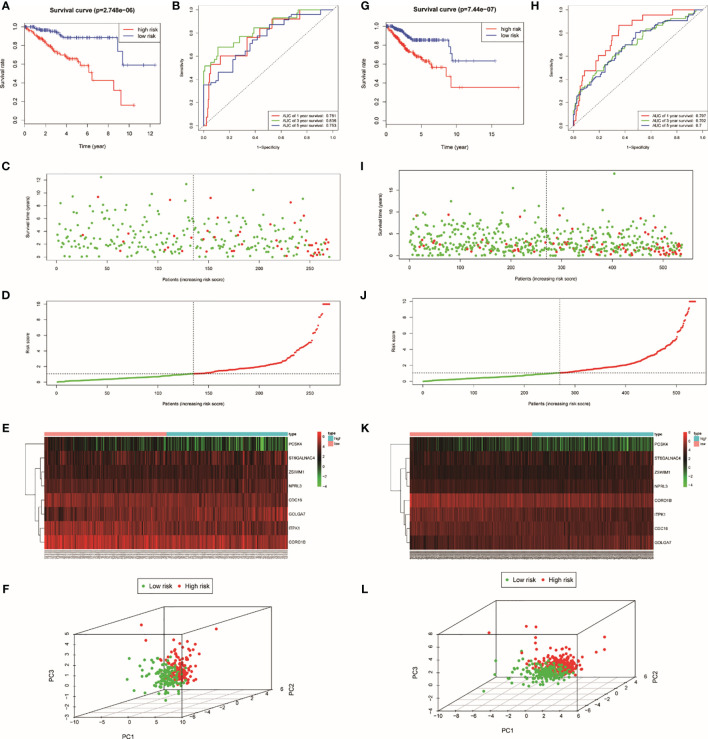
Identification of TRRS in the train set **(A–F)** and the entire set **(G–L)**. **(A, G)** Kaplan–Meier curve analysis of overall survival of cervical cancer patients in high- and low-risk groups. **(B, K)** Time-dependent ROC curves analysis. Risk score distribution **(C, I)**, survival status **(D, J)**, and genes expression patterns **(E, K)** for patients in high- and low-risk groups by the TRRS. **(F, L)** Principal component analysis.

In order to estimate the prognostic performance of DE genes, univariate Cox regression analysis was employed to the 251 genes (**Supplementary Table S2**). In the train set, 12 genes were closely related to the OS of UCEC patients (*p* < 0.05). Twelve genes were analyzed by LASSO, and eight of them were selected (**Supplementary Figures S3A, B**). After this, a multivariate Cox regression analysis was implemented, and five genes had significant statistical correlation with the hazard ratio of UCEC patients in the train set (**Supplementary Figure S3C**). We utilized these genes to establish the TRRS. Eight genes were weighted by relative coefficient, and the formula is as follows: risk score = (−0.070*CDC16) + (0.010*ZSWIM1) + (−0.045*ITPK1) + (0.099*NPRL3) + (0.274*GOLGA7) + (0.022*ST6GALNAC4) + (−0.201*PCSK4) + (−0.026*CORO1B). These eight genes were associated with high risk, including ZSWIM1 [hazards ratio (HR) =1.104(1.009–1.208), *p* = 0.031], NPRL3 [HR = 1.104(1.023–1.191), *p* = 0.010), GOLGA7 [HR = 1.027(0.995–1.061), *p* = 0.096], ST6GALNAC4 [HR = 1.022(0.998–1.046), *p* = 0.072], CDC16 [HR = 0.932(0.881–0.996), *p* = 0.015], ITPK1 [HR = 0.956(0.922–0.991), *p* = 0.015], PCSK4 (HR = 0.818[0.656–1.020], *p* = 0.072], and CORO1B [HR = 0.974(0.951–0.998), *p* = 0.033) (**Table 1**). The risk score of samples in the train set were computed according to the above formula. People in the train set were split into high-risk group (n = 134) and low-risk group (n = 135) on the basis of their median-risk score. The comparison showed that there exists a significant difference in the OS of different groups (*p* = 2.74e−06, log-rank test) (**Figure 3A**). The AUC for the signature of OS in 5 years is 0.753, and it is 0.836 in 3 years and 0.781 in 1 year (**Figure 3B**). We sort the patient’s risk scores, and their distribution is shown in **Figure 3C**. The living condition of UCEC patients is shown in the dot plot (**Figure 3D**). Gene expression pattern between the two groups of patients with different prognosis is presented in the heatmap expression (**Figure 3E**). Low- and high-risk patients showed significant difference using principal component analysis (PCA) (**Figure 3F**).

**Table 1:** Multivariate Cox regression eight genes weighted by their relative coefficient.

Gene	HR (95% CI)	*p* value
*CDC16*	0.93 (0.88, 0.99)	0.01
*ZSWIM1*	1.10 (1.01, 1.21)	0.03
*ITPK1*	0.96 (0.92, 1.00)	0.02
*NPRL3*	1.10 (1.02, 1.19)	0.01
*GOLGA7*	1.03 (1.00, 1.06)	0.10
*ST6GALNAC4*	1.02 (1.00, 1.05)	0.07
*PCSK4*	0.81 (0.66, 1.02)	0.07
*CORO1B*	0.97 (0.95, 1.00)	0.03

### The Relationships Between TRRS Expression and Clinical Factors

We achieved the expression profiles of TRRS from the TCGA. GOLGA7, ITPK1, and ST6GALNAC4 genes had lower expression in tumor tissues than in healthy persons (*p* < 0.05) by means of Wilcoxon signed-rank test (**Supplementary Figure S4A**). After that, qRT-PCR has been used to compare the difference in TRRS expression between normal and tumor tissue (**Supplementary Figure S4B**). The results showed that the expression levels of CORO1B, GOLGA7, PCSK4, ST6GALNAC4, and ZSWIM1 in normal tissues were significantly higher than those in tumor tissues, which were similar to the trends detected in the TCGA dataset. Then, the stratified studies were utilized to see whether the genes have different expression level in different clinical characteristics (**Supplementary Figure S5**). It is not hard to find that most of the eight TRRS have lower expression levels in advanced stage. The result showed that higher expression of GOLGA7 [HR =2.28(1.46–3.56), *p* < 0.001] and ZSWIM1 [HR = 1.7(1.09–2.67), *p* = 0.011] were combined with poorer OS of UCEC patients; however, CORO1B [HR = 0.51(0.33–0.79), *p* = 0.001], CDC16 [HR = 0.52(0.33–0.81), *p* = 0.001], PCSK4 [HR = 0.51 (0.32–0.81), *p* = 0.001], and ITPK1 [HR = 0.52(0.34–0.8), *p* = 0.002] expression level had a positively correlated with overall survival (**Supplementary Figure S6**).

### Genome-Wide Analysis of TRRS

**Figure 4:**
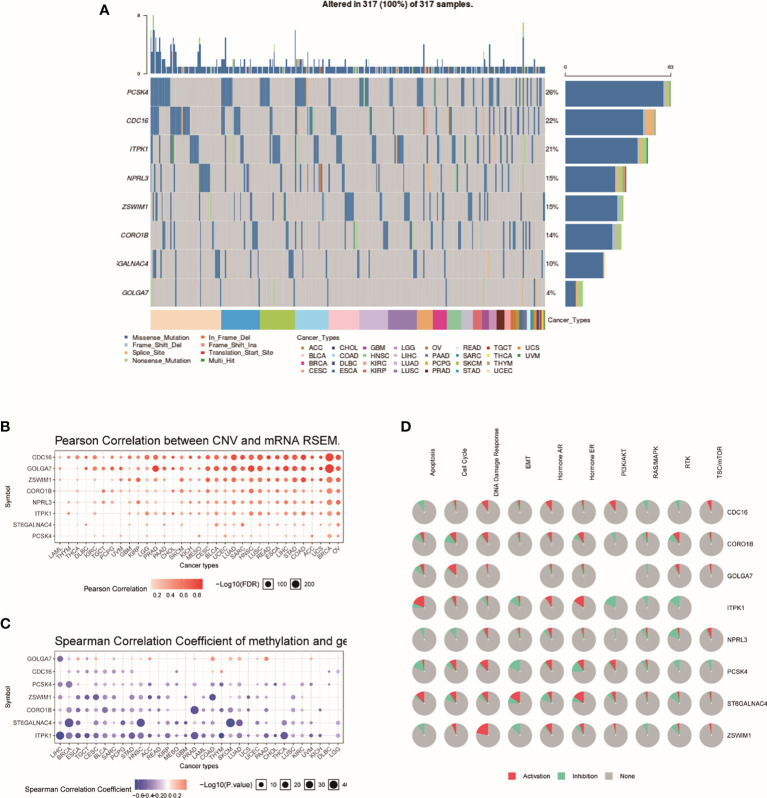
Genome-wide analysis of TRRS. **(A)** An oncoplot, also known as a waterfall plot, shows the mutation distribution of the key genes in a gene set and a SNV classification of SNV types (including missense mutation, frame shift deletion, nonsense mutation, etc.). All selected cancers’ samples are shown together. Side barplot and top barplots show the number of variants in each sample or gene. **(B)** This figure demonstrates the relation between CNV and gene expression. The red bubbles represent a positive correlation, which means that when gene has a high frequency of CNV, the gene expression will become upregulated. The deeper the color, the higher the correlation. The size of the point represents statistical significance; the greater the size, the more it is statistically significant. **(C)** The bubble plot shows the Spearman correlation coefficient of methylation and gene expression. Blue points represent a methylation downregulation in tumors and red points a methylation upregulation in tumors, in which the deeper the color, the greater the difference. The size of the point represents statistical significance; the greater the size, the more the significance. **(D)** This plot displays the global activity of genes in selected cancer types.

Genome-wide analysis of TRRS was carried out by utilizing the GSCALite. The results indicated that PCSK4 was the gene with the highest mutation frequency, followed by CDC16 and ITPK1, while GOLGA7 had the lowest (**Figure 4A**). The expression of the eight genes in TRRS had a positive correlation with copy number variations (CNVs), which is performed in the bubble diagram (**Figure 4B**). CNV frequency was positively correlated with gene expression. Spearman correlation coefficient between methylation and gene expression was performed in the methylation difference bubble chart. We found that the GOLGA7, PCSK4, ZSWIM1, ST6GALNAC4, and ITPK1’s methylation was downregulated in UCEC (**Figure 4C**). This means that methylation had a significant effect on gene expression. The exploration of pathway activity showed that ZSWIM1, PCSK4, CDC16, and CORO1B had positive correlations to DNA damage response pathway activation; ITPK1, NPRL3, PCSK4, ST6GALNAC4, and ZSWIM1 were related to the RTK pathway inhibition; and ITPK1 activated the apoptosis and inhibit PI3K/AKT pathway significantly (**Figure 4D**).

### Verifying the Predictive Capability of the Eight TRRS

For the purpose of exploring the predictive power of TRRS, we constructed an entire set. In the entire set, the risk score was computed by using the median risk score. Each patient in the entire set was split into two groups according to the risk scores. High-risk groups have 267 cases and low-risk groups have 270. There was significant statistical distinction between the two groups’ Kaplan–Meier survival curves (**Figure 3G**, P =7.44e-07). In the entire set, the AUC was 0.797 in 1 year, 0.702 in 3 years, and 0.7 in 5 years (**Figure 3H**). The situation of risk score, survival status, and expression of eight TRRS in the entire set are performed in **Figures 3I–K**. Principal component analysis also showed a discrepancy between the groups (**Figure 3L**).

### Using Nomogram to Predict the Survival Rates

**Figure 5:**
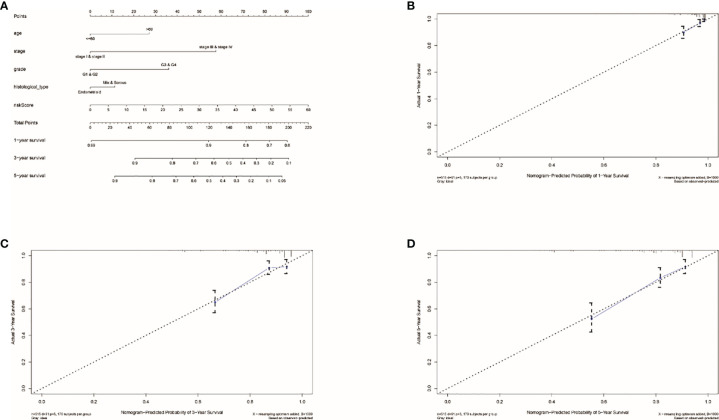
Results of nomogram prediction. **(A)** An immune nomogram for predicting the survival rates of UCEC patients in 1, 3, and 5 years. The calibration curve of the constructed nomogram of 1- **(B)**, 3- **(C)**, and 5-year **(D)** survival.

By using the known risk score and some clinical characteristics, multivariate logistic regression was applied to structure a nomogram that may predict the survival rates of UCEC patients accurately. Age, stage, grade, histological type, and risk score were considered predictors of survival rates, which were combined into the nomogram (**Figure 5A**). It showed that risk score is the most influential factors of the nomogram total score. The calibration curve of the constructed nomogram (**Figures 5B–D**) performed that the survival rates predicted by TRRS was almost consistent with the actually survival rates we observed. This means the TRRS has good clinical practicability.

### Clinical Factors and Risk Score Will Affect the Prognosis

**Figure 6:**
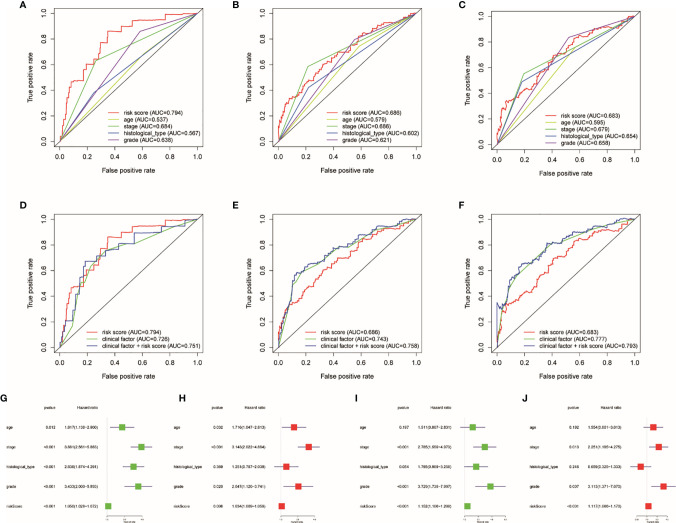
Influence of clinical factors and risk score on prognosis. Clinical factor and risk score AUC of 1- **(A, D)**, 3- **(B, E)**, and 5-year **(C, F)** survival rates. Uni- and multivariate Cox regression analyses in the entire **(G, H)** and train sets **(I, J)**.

AUC was employed to calculate the prognostic capability of clinical factor and risk score. The higher the AUC, the more precise the TRRS. Risk score and four clinical factors are shown on **Figures 6A–C**. The AUCs of the clinical factors combined with the risk score of 1-, 3- and 5-year survival rates were 0.751, 0.758, and 0.758, respectively (**Figures 6D–F**). The result showed that using the risk score combined with clinical characteristics to evaluate the prognosis resulted in high sensitivity and specificity. After that, Cox regression analyses including univariate and multivariate regression were applied to calculate the prognosis capacity of risk score and clinical factors. As performed in **Figures 6G, H**, the outcome of univariate cox regression analysis in the entire set showed that stage (HR, 3.881; 95%CI, 2.561–5.883, *p* < 0.001), histological type (HR, 2.836; 95%CI, 1.874–4.291; *p* < 0.001), and risk score (HR, 1.050; 95%CI, 1.028–1.072; *p* < 0.001) were related to UCEC prognosis. However, the outcome of multivariate Cox regression indicated that stage (HR, 2.836; 95%CI, 1.874–4.291; *p* < 0.001) is an independent prognostic factor of UCEC. Besides, the stage (HR, 2.785; 95% CI, 1.559–4.973; *p* < 0.001), grade (HR, 3.729; 95% CI, 1.739–7.997; *p* < 0.001), and risk score (HR, 1.152; 95%CI, 1.106–1.200; *p* < 0.001) were related to the prognosis of UCEC. However, in the train set, only the risk score (HR, 1.171; 95%CI, 1.066–1.170; *p* < 0.001) was independently correlated with the prognosis of UCEC patients (**Figures 6I, J**). Then we used prognostic stratification to analyze the function of risk score to judge the prognosis of UCEC. The result indicated that with different clinical characteristics (age, stage, grade, and endometrial histological type), UCEC patients with different risks have different overall survival rates (**Supplementary Figure S7**). This result also proved that risk score can affect the prognosis of UCEC patients independently.

### GSEA and ESTIMATE and ssGSEA Analysis

**Figure 7:**
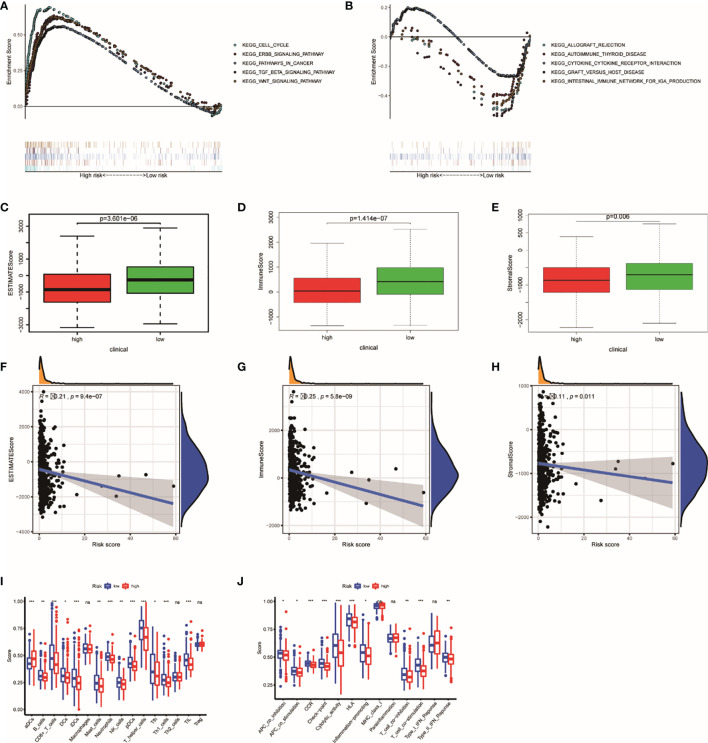
The results of GSEA and ESTIMATE and ssGSEA analysis. Gene set enrichment analysis based on KEGG of high- and low-risk groups **(A, B)**. The boxplot showed the difference in ESTIMATE score **(C)**, immune score **(D)**, and stromal score **(E)** calculated using ESTIMATE algorithm between the two groups. The plot showed the relationships between ESTIMATE score **(F)**, immune score **(G)**, and stromal score **(H)**. The difference in each immune cell **(I)** and immune function **(J)** calculated by ssGSEA method between high- and low-risk groups. *P < 0.05; **P < 0.01; ***P < 0.001. ns, not significant.

Through GSEA, we concluded that the high risk is related to tumor-related pathways (**Figure 7A**), which can explain why high-risk groups have poor prognosis. Low risk in patients is associated with the pathways that related to immunity (**Figure 7B**). Therefore, we use ESTIMATE and ssGSEA analysis to verify the difference in immunological status between the groups. The immune score, stromal score, and estimate score were achieved by ESTIMATE algorithm through R “estimate” package (**Figures 7C–H**). The study on the association between risk score and immunity based on ssGSEA also confirmed that the risk score was negatively correlated with its immune ability (**Figures 7I, J**).

### The Relationship Between the TRRS and Immune Response in UCEC

**Figure 8:**
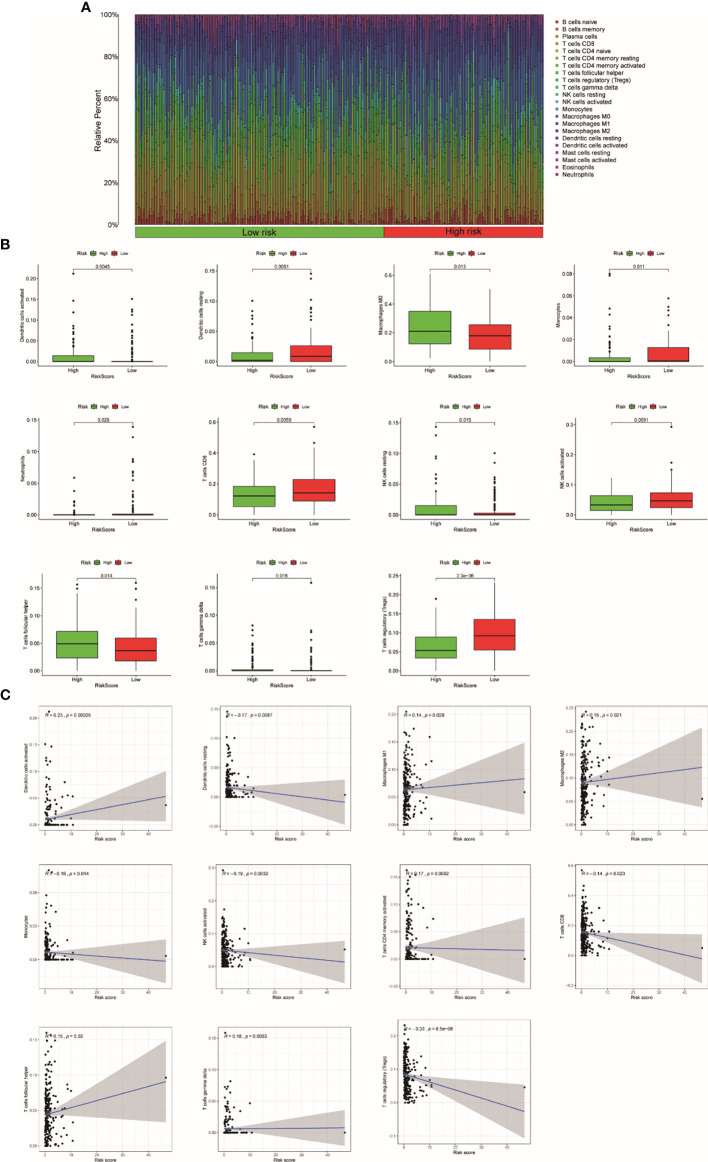
The relationship between the TRRS and immune response. **(A)** Relative percentage of each type of immune cell in 253 EC samples from TCGA cohort **(B)**. The difference in immune response between high- and low-risk groups. **(C)** Relationship between immune response and risk score.

According to our previous studies, we comprehensively assess the values of 22 immune cells by using CIBERSORT. **Figure 8A** shows the results we got from 251 UCEC samples. The result showed that dendritic cells activation, macrophage M0, resting NK cells, T-follicular helper cells, and gamma delta T cells were obviously stronger in the high-risk group. Resting dendritic cells, monocytes, neutrophils, CD8 T cells, activating NK cells, and Tregs were obviously higher in low-risk patients (**Figure 8B**). The results of the relationship analysis were consistent with this. Activated dendritic cells (R = 0.23, *p* < 0.001), macrophage M1 (R = 0.14, *p* = 0.029), and macrophage M2 (R = 0.15, *p* = 0.021), T follicular helper cells (R = 0.15, *p* = 0.020), and gamma delta T cells (R = 0.18, *p* = 0.005) were positively associated with the risk score. While resting dendritic cells (R = −0.17, *p* = 0.009), monocytes (R = −0.16, *p* = 0.014), activating NK cells activated (R = −0.19, *p* = 0.003), CD8 T cells (R = −0.14, *p* = 0.023) were positively associated with the risk score. Specially, the risk scores were strongly negatively related to the expression of Tregs (R = −0.33, *p* = 8.5e−08). This is consistent with the previous results (**Figure 8C**).

### IPS and Immune Checkpoints

**Figure 9:**
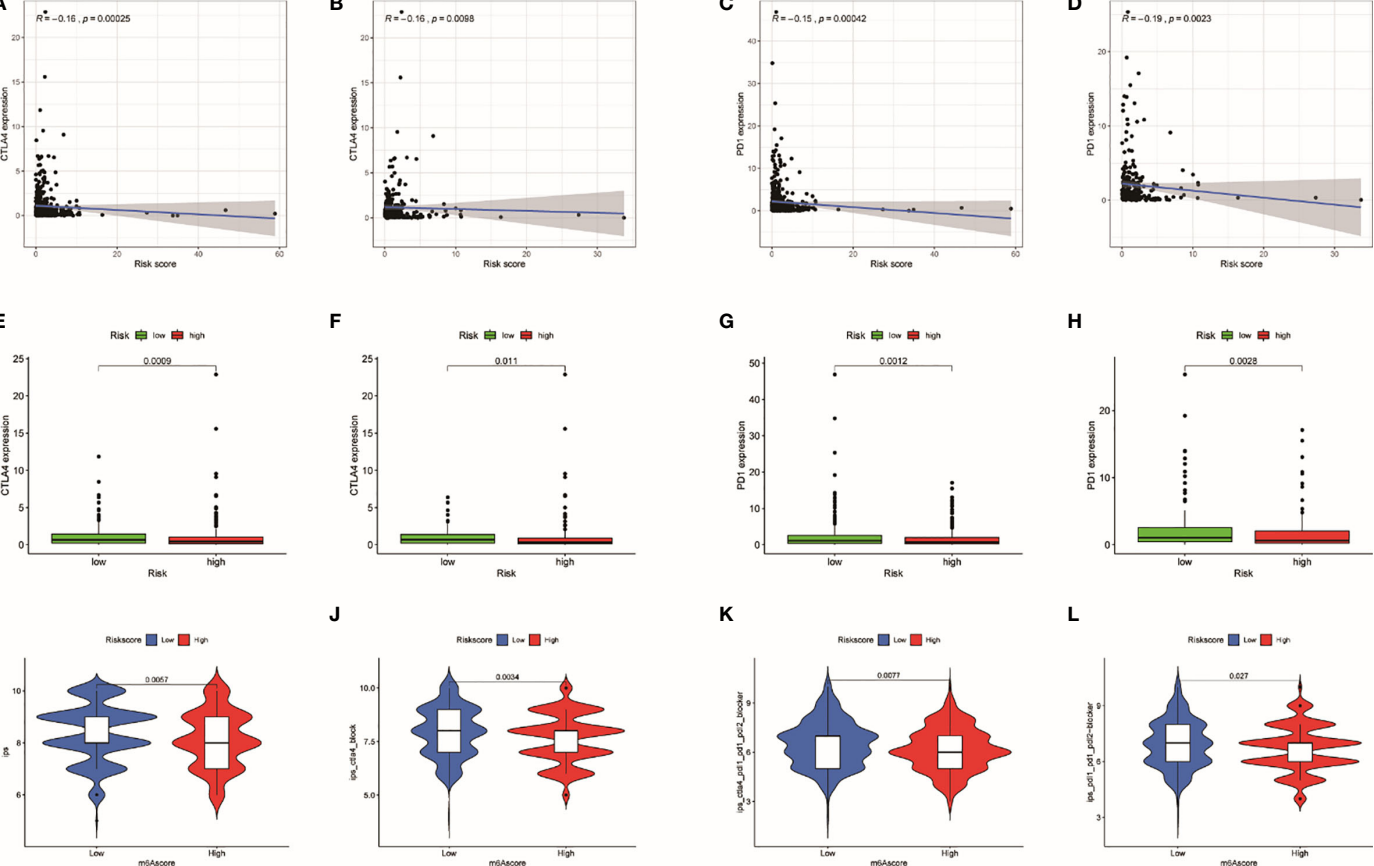
The relationship of risk score with immune checkpoints. **(A)** Relationship between risk score with CTLA-4 expression in the entire set. **(B)** Relationship between risk score with CTLA-4 expression in the train set. **(C)** Relationship between risk score with PD1 expression in the entire set. **(D)** Relationship between risk score with PD1 expression in the train set. **(E)** Differences in CTLA-4 expression between high- and low-risk patients in the entire set. **(F)** Differences in CTLA-4 expression between high- and low-risk patients in the train set. **(G)** Differences in PD1 expression between high- and low-risk patients in the entire set. **(H)** Differences in PD1 expression between high- and low-risk patients in the train set. **(I–L)** The association between IPS and the TRRS in UCEC patients.

Immune checkpoints (CTLA4 and PD1) are able to assess the response of patients to immunotherapy. The result showed that the expression of CTLA4 (entire set: R = −0.16, *p* = 0.00025; train set, R = −0.16, *p* = 0.0098) and PD1 (entire set: R = −0.15, *p* = 0.00042; train set: R = −0.19, *p* = 0.0023) were negatively correlated with TRRS in both train set and entire set (**Figures 9A–D**). In addition, patients in lower risk presented higher gene expression of CTLA4 and PD1 in both train set and entire set (*p* < 0.05) (**Figures 9E–H**). Consequently, we supposed that immune checkpoints related to Tregs might be used in immunotherapy of UCEC. In this study, we further analyzed the correlation between IPS and TRRS in UCEC. The IPS, IPS-CTLA4, IPS-CTLA4/PD-L1/PD1/PD-L2, and IPS-PD1/PD-L1/PD-L2 scores were employed to assess the probability of ICI. Low-risk patients had significantly higher scores (**Figures 9I–L**: IPS, *p* < 0.001; IPS-CTLA4, *p* = 0.003; IPS-CTLA4/PD-L1/PD1/PD-L2, *p* = 0.008; and IPS-PD1/PD-L1/PD-L2, *p* = 0.027). Consequently, we inferred that patients with low risk are more likely to trigger an immune response.

### The TRRS and Mutation Profile

**Figure 10:**
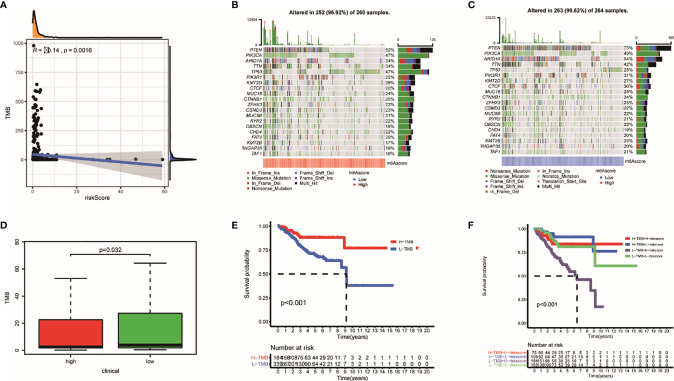
The mutation profile and TMB among low- and high-risk groups. **(A)** The relationship between TMB and TRRS. **(B, C)** Mutation profile of low- and high-risk groups. **(D)** The relationship between the TRRS and TMB. **(E, F)** The association of TMB and prognosis in TCGA UCEC dataset.

Tumor burden has always been an important factor affecting immunotherapy (35). In our study, TMBs are negatively correlated with TRRS (**Figure 10A**). Genes that had the most frequent mutation in each groups are shown in **Figures 10B, C**. Low-risk patients had heavier TMB (*p* = 0.032) (**Figure 10D**). Furthermore, survival probability of lower TMB patients was significantly lower than those of patients with higher TMB (**Figure 10E**). Meanwhile, patients with lower tumor mutational burden and high risk had the lowest survival probability compared with other groups (*p* < 0.001) (**Figure 10F**).

### Relationship Between TRRS and Chemotherapy Sensitivity

**Figure 11:**
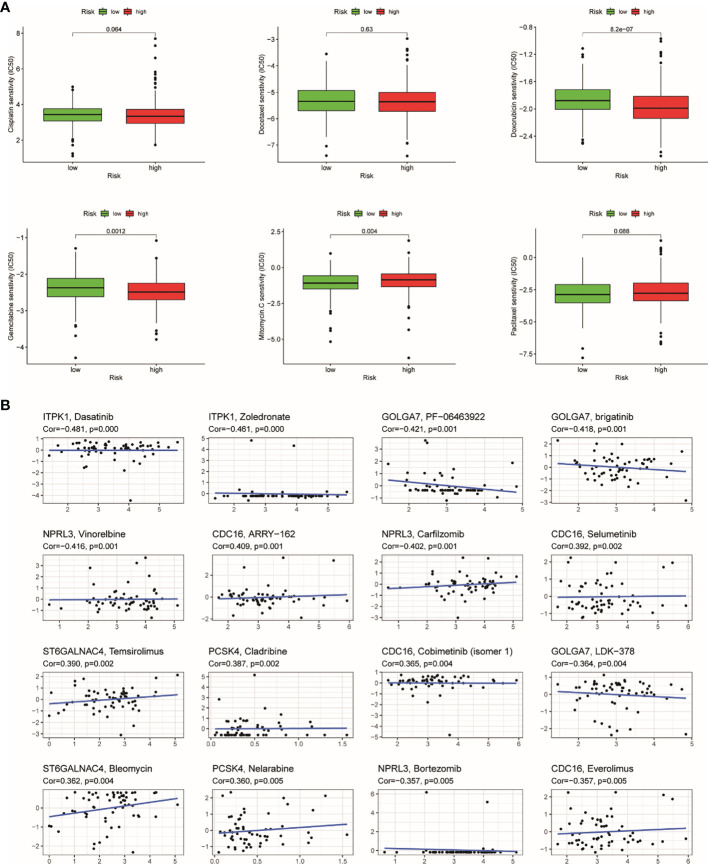
The results of chemosensitivity analysis. **(A)** Association between the risk score and chemosensitivity in UCEC. The box plots of the estimated IC50 for cisplatin, docetaxel, doxorubicin, gemcitabine, methotrexate, and paclitaxel were shown in the two groups. **(B)** Scatter plot of relationship between prognostic gene expression and drug sensitivity.

Recently, chemotherapy is an ordinary treatment for UCEC; we analyzed the response of two groups to six chemotherapeutic drugs including cisplatin, docetaxel, doxorubicin, gemcitabine, methotrexate, and paclitaxel. We calculated IC50 for each samples using the TRRS. The results showed that half of those drugs have significant distinction between the groups. Doxorubicin (*p* = 0.001) and gemcitabine (*p* = 0.004) have higher sensitivity in the low-risk group, which indicated that chemotherapeutic drugs may have better curative effect in the high-risk group (**Figure 11A**). After that, we further analyzed the relationship between expression of genes in TRRS and chemotherapy sensitivity. The outcome indicated that seven genes were strongly correlated to the sensitivity of some chemotherapeutic drug (*p* < 0.01) (**Figure 11B**). For example, GOLGA7, ITPK1, and NPRL3 were correlated with increased drug resistance of cancer cells to Dasatinib, Zoledronate, PF-06463922, Brigatinib, LDK-378, Vinorelbine, Carfilzomib, and Bortezomib, respectively. Meanwhile, increased expression of ST6GALNAC4 and PCSK4 was related to more sensitivity of tumor cells to a number of chemotherapy drugs like Temsirolimus, Bleomycin, Nelarabine, and Cladribine. In addition, CDC16 was positively correlated with ARRY-162 and Selumetinib but was negatively correlated with Everolimus. The mechanism needs further study.

### Immunoassay for ZSWIM1

**Figure 12:**
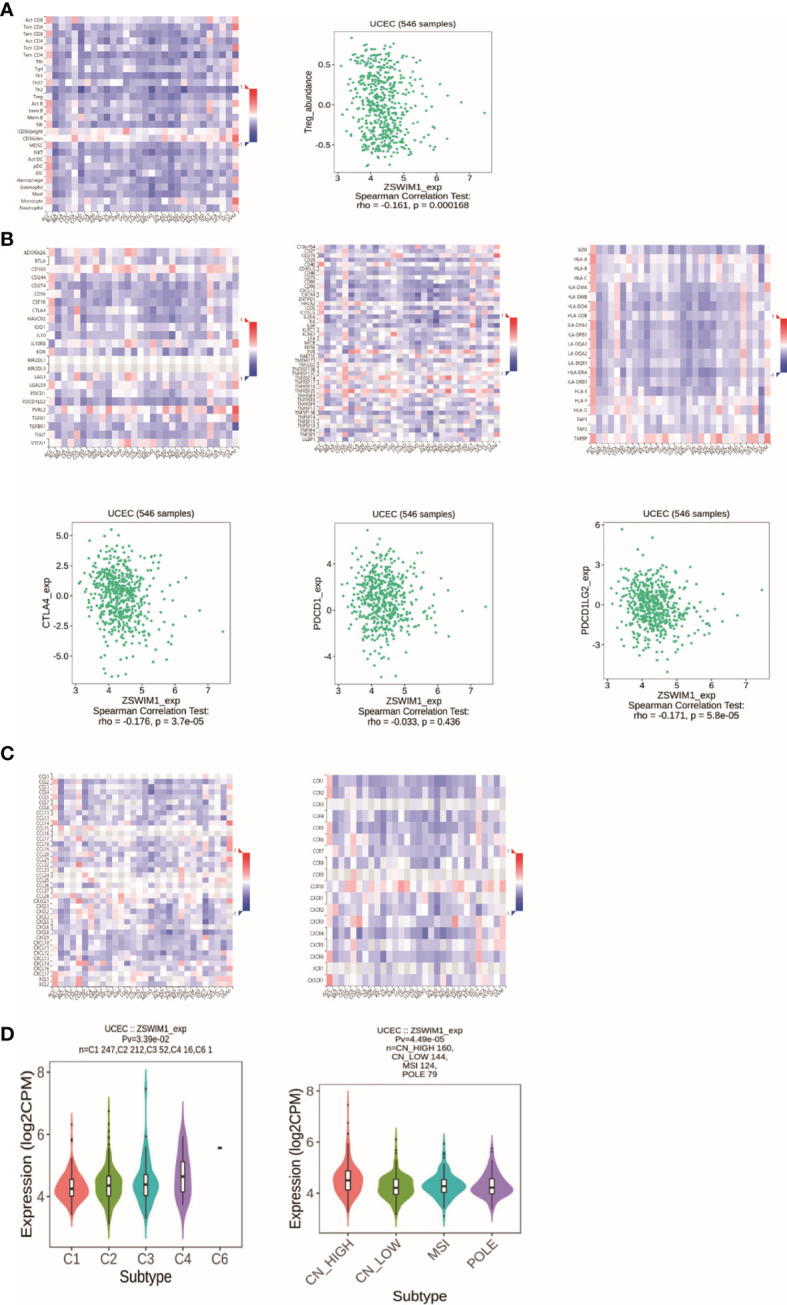
The results of immunoassay for ZSWIM1. **(A)** The landscape of relationship between ZSWIM1 expression and TILs in different types of cancer (red is positive correlation, and blue is negative correlation). **(B)** The relationship between ZSWIM1 expression and immunomodulator. **(C)** The relationship between ZSWIM1 expression and chemokine. **(D)** The relationship between ZSWIM1 expression and subtype.

Among the eight genes, only the expression of ZSWIM1 was consistent with the prognosis, so we chose ZSWIM1 for further analysis. Tumor-infiltrating lymphocytes (TILs) can undertake the role of an independent predictor in several cancers. Consequently, TISIDB was applied to analyze the relationship between TILs and ZSWIM1expression. The correlation between ZSWIM1 expression and TILs in different descriptions of cancer is performed in **Figure 12A**. The expression of ZSWIM1 was negatively correlated with Tregs in UCEC (R = −0.161, *p* = 0.000168). Immunopotentiators, immunosuppressants, MHC molecules (**Figure 12B**), chemokines, and receptors (**Figure 12C**) had a negative relationship with the expression of ZSWIM1. There exist significant distinction in the expression of ZSWIM1 among different immune and molecular subtypes of UCEC. The relationship between ZSWIM1 expression and human cancer immune subtypes is shown in **Figure 12D**. Specifically, the expression of ZSWIM1 in wound healing, interferon gamma (IFN-γ) dominance, inflammation, lymphocyte depletion, and transforming growth factor alpha (TGF-α) was increased in turn. However, it was negatively correlated with molecular subtypes.

## Discussion

UCEC is a common gynecological malignant tumor (36) with low survival rate and poor prognosis (37–39). However, if the disease is correctly diagnosed in the early stage, the 5-year survival rate can be as high as 90% (40). At present, the treatment of EC is based on surgical operation (41), postoperative radiotherapy, and chemotherapy, which can be performed according to the type and stage of tumor (42). However, patients in same clinical stage may manifest different clinical characteristics, indicating that it is insufficient to estimate the prognosis of UCEC according to the traditional clinicopathological staging (7). In recent years, CA125 and HE4 have been used as serum markers of UCEC. However, the accuracy of serum prediction is relatively low (43). Clinical trials of UCEC patient showed that the effective rate of mammalian target of rapamycin (mTOR) inhibitors treatment was <10% (44). Immunotherapy has replaced traditional radiotherapy and chemotherapy as a remedy method for cancer. Especially, CTLA-4, PD-1, and PD-L1 antibodies have curative effect on tumor treatment (45). In this study, The Cancer Genome Atlas (TCGA) database was employed to construct TRRS for patients with UCEC, which is expected to be used to monitor prognosis and immunotherapy response in UCEC.

In the immunotherapy of different kinds of cancer, blocking immune checkpoint has shown a wide range of effects (46). Antibodies against checkpoint molecule, namely, CTLA4, PD1, and PD-L1 have shown clinical efficacy and persistence in more than 15 kinds of human malignant caners (47, 48). This has been approved by the Food and Drug Administration (FDA) as a treatment strategy for a variety of cancers. In particular, anti-PD-1 combination therapy can be applied as a remedy for advanced melanoma, which has good curative effect compared with any single drug therapy (49).

At the beginning, CIBERSORT was employed to assess the proportion of T cells, which indicated that the more advanced the clinical stage and grade, the lower the expression of Tregs. This means that TRRS is a tumor suppressor gene. By using the WGCNA algorithm and correlation analysis, the red module was selected as the most relevant module. GO and KEGG enrichment analysis showed that the red module has the closest relationship with Tregs. Univariate, LASSO, and multivariate Cox regression were applied to calculate prognostic significances in risk score and some clinical factors.

Genome-wide analysis of these eight genes indicated that these high frequency mutations and methylation had close relationships with gene expression, and they were involved in regulating the activity of injury response pathway. The high expression of these genes in tumor samples and early clinical stage indicates their potential use as biomarkers. The outcomes of qRT-PCR were similar to the trends detected in the TCGA dataset, which confirmed the prediction ability of the model to a certain degree. The AUC of the risk score showed the high sensitivity and specificity of TRRS. The AUCs of the risk score merged with clinical characteristics were higher, which suggests that using the TRRS together with clinical factors, we can give patients a better treatment guidance. In line with previous studies, the results of this study showed that age, stage, and grade were also connected with the prognosis of patients with UCEC. The nomogram shows that the TRRS can accurately assess the prognosis of UCEC (7). Studies on several common chemotherapeutic drug reactions have found that low-risk patients had higher sensitivity to adriamycin and gemcitabine; furthermore, the expression of CTLA-4, PD1, and PDL1 was higher in the low-risk group, which means that chemotherapeutic drugs could have better efficacy in low-risk patients. TMB was used to assess the ability of TRRS to identify patients who have higher response to ICI (35). TMB was higher in low-risk groups with UCEC. This means that low-risk patients have the possibility to be identified by immune cells and can benefit more from immunotherapy. In addition, the survival probability of low TMB cases was significantly lighter than that of cases with high TMB.

Eight genes were selected for further analysis and had been used as prognostic markers in other diseases. CDC16 has a connection with multiple neurodevelopmental disorders (50, 51). It has been found that CDC16 has potential therapeutic function in melanoma (52). ITPK1 may serve as biomarkers for GC pathogenesis (53, 54). The expression of PCSK4 mRNA is decreased in non-islet-cell tumor hypoglycemia (NICTH) patients, which has been demonstrated to be related to serum big IGF2 increase (55). Coronin 1B (Coro1B) is one of the actin binding proteins that can regulate platelet-derived growth factor (PDGF)-induced vascular smooth muscle cell (VSMC) migration, suggesting a new therapeutic target for vasculopathies (56). ZSWIM1 can be used as a biomarker of T helper cell differentiation (57). Nitrogen permease regulator-like 3 took part in the construct of GATOR1 complex (**Supplementary Figure S1**), which can regulate the mTOR pathway (58). Chang Soo Ryu found that NPRL3 is a common biomarker for ischemic stroke (59). Linghui Zhou found that rs11337 (G > T) in GOLGA7 is related to survival of glioma patients (60). ST6GALNAC4 expression is related to glycosphingolipids synthesis, which has a connection with breast cancer (61, 62). By using GSEA, tumor-related pathways like ERBB, TGF-BETA, and WNT were significantly enriched; these pathways are deemed to be associated with tumor, which could be used as novel therapeutic targets (63–65). Immune-related responses were significantly enriched in low-risk patients, which further validated the difference in immune status between the two risk groups. Besides, high-risk cases had higher fractions of monocytes, NK cells, CD8 T cells, neutrophils, and Tregs. Researchers have confirmed that NK cells, CD8 T cells, neutrophils, and Tregs were significantly associated with survival of UCEC patients (66).

Genome-wide analysis of the genes showed that the mutation frequency of PCSK4 was the highest. The expression of the TRRS was positively correlated with CNV. Besides, the methylation of GOLGA7, PCSK4, ZSWIM1, ST6GALNAC4, and ITPK1 was downregulated. DNA methylation is an epigenetic mechanism to control the expression of oncogenic or tumor-suppressive genes. Scientists have studied the potential effectiveness of some methylated biomarkers in predicting cancer prognosis (67). However, there has been seldom studies on these genes’ methylation in UCEC, so it makes sense to do further research. Further study on ZSWIM1 based on TISIDB showed that the expression level of ZSWIM1 in different immune subtypes and molecular subtypes of UCEC was significantly different. The immunosuppressant was effective on ZSWIM1 gene. Therefore, ZSWIM1 can be considered as a target for UCEC treatment.

Previous studies have also found gene prognostic markers closely related to UCEC (68–70), but it is the first time to construct a model in Treg cells to predict the prognosis of UCEC. AUC is a crucial standard to judge whether a prediction model has good discrimination. In another article that also identified a signature for predicting the prognosis of patients with UCEC, the AUC of the prognostic model using 10 immune genes was 0.756 (7). In this study, the AUC for the signature that we constructed in 1 year is 0.781, and it is 0.836 in 3 years and 0.753 in 5 year. It is higher than that of a previous study that showed high sensitivity and specificity of our model, suggesting that the model has a better ability to predict the probability of disease occurrence. Nonetheless, this study still has some deficiencies. The conclusion of our study is mainly on account of bioinformatics analysis, and further clinical research is needed. Furthermore, risk factors of UCEC, such as obesity and smoking, were not discussed in this study.

## Conclusion

All in all, through a series of bioinformatics analysis, we constructed a TRRS as potential biomarkers and targets for immunotherapy of UCEC. Low-risk patients had better prognosis and higher response rate to ICI. In the future, TRRS is expected to help predict prognosis and assess the efficacy of immunotherapy for UCEC patients, which can provide individualized treatment.

## Data Availability Statement

The datasets presented in this study can be found in online repositories. The names of the repository/repositories and accession number(s) can be found in the article/**Supplementary Material**.

## Author Contributions

JB, JL, and RG conceived the study and participated in the study design, performance, and manuscript writing. JL, SY, and FS conducted the bioinformatics analysis. ZZ, MY, SN, and LC revised the manuscript. All authors contributed to the article and approved the submitted version.

## Conflict of Interest

The authors declare that the research was conducted in the absence of any commercial or financial relationships that could be construed as a potential conflict of interest.

## Publisher’s Note

All claims expressed in this article are solely those of the authors and do not necessarily represent those of their affiliated organizations, or those of the publisher, the editors and the reviewers. Any product that may be evaluated in this article, or claim that may be made by its manufacturer, is not guaranteed or endorsed by the publisher.

## Supplementary Material

Supplementary Figure 1 Soft-thresholding parameters of WGCNA. **(A)** Analyze the scale-free fit index for various soft-thresholding parameters. **(B)** Analyze the mean connectivity for various soft-thresholding parameters. **(C)** Histogram of connectivity distribution when β=6. **(D)** Check of scale-free topology when β=6.

Supplementary Figure 2 GO and KEGG analysis **(A)** Top ten biological process related to key gene using GO analysis. **(B) **Top ten cellular component related to key genes using GO analysis. **(C) **Top ten molecular function related to key genes using GO analysis. **(D) **Top ten human pathway related to key genes using KEGG analysis.

Supplementary Figure 3 LASSO COX regression and multivariate Cox analysis. **(A, B)** LASSO COX regression for OS of TRRS in the TCGA cohort. **(C)** Forest plot illustrating the multivariate Cox model results of eight gene related to Tregs.

Supplementary Figure 4 Comparison of the expression of the eight genes. **(A) **Expression levels of the eight genes between EC samples and normal tissues evaluated by means of Wilcoxon signed-rank test. **(B) **Expression levels of the eight genes between normal tissues and tumor tissue evaluated by using qRT-PCR.

Supplementary Figure 5 The relationships between gene expression and clinical characteristic.

Supplementary Figure 6 The association of expression of eight gene and OS in TCGA UCEC dataset. **(A) **GOLGA7; **(B) **CORO1B; **(C) **CDC16; **(D) **ZSWIM1; **(E)**PCSK4; **(F)**ITPK1.

Supplementary Figure 7 Overall survival result between patients with high and low risk. **(A) **age>60; **(B) **age<60; **(C) **grade G1&G2; **(D)** grade G3&G4; **(E)**histological-type Mixed &Serous; **(F) **Stage I& Stage II; **(G)** Stage III& Stage IV.
