# Supplementary figures and images for: Development and Clinical Validation of Novel 8-Gene Prognostic Signature Associated With the Proportion of Regulatory T Cells by Weighted Gene Co-Expression Network Analysis in Uterine Corpus Endometrial Carcinoma
Source: Front Immunol. 2021 Dec 14;12:788431. doi: 10.3389/fimmu.2021.788431 (PMC8712567; doi:10.3389/fimmu.2021.788431)

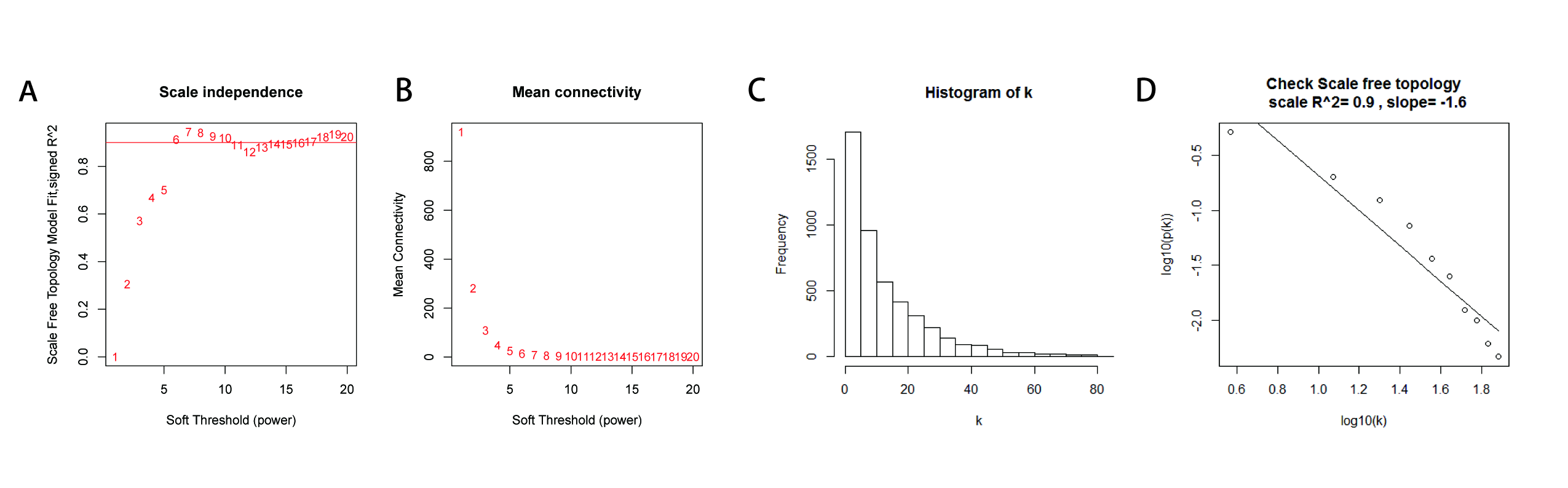

Supplement: Supplementary Figure 1 — Soft-thresholding parameters of WGCNA. (A) Analyze the scale-free fit index for various soft-thresholding parameters. (B) Analyze the mean connectivity for various soft-thresholding parameters. (C) Histogram of connectivity distribution when β=6. (D) Check of scale-free topology when β=6. [file Image_1.tif]

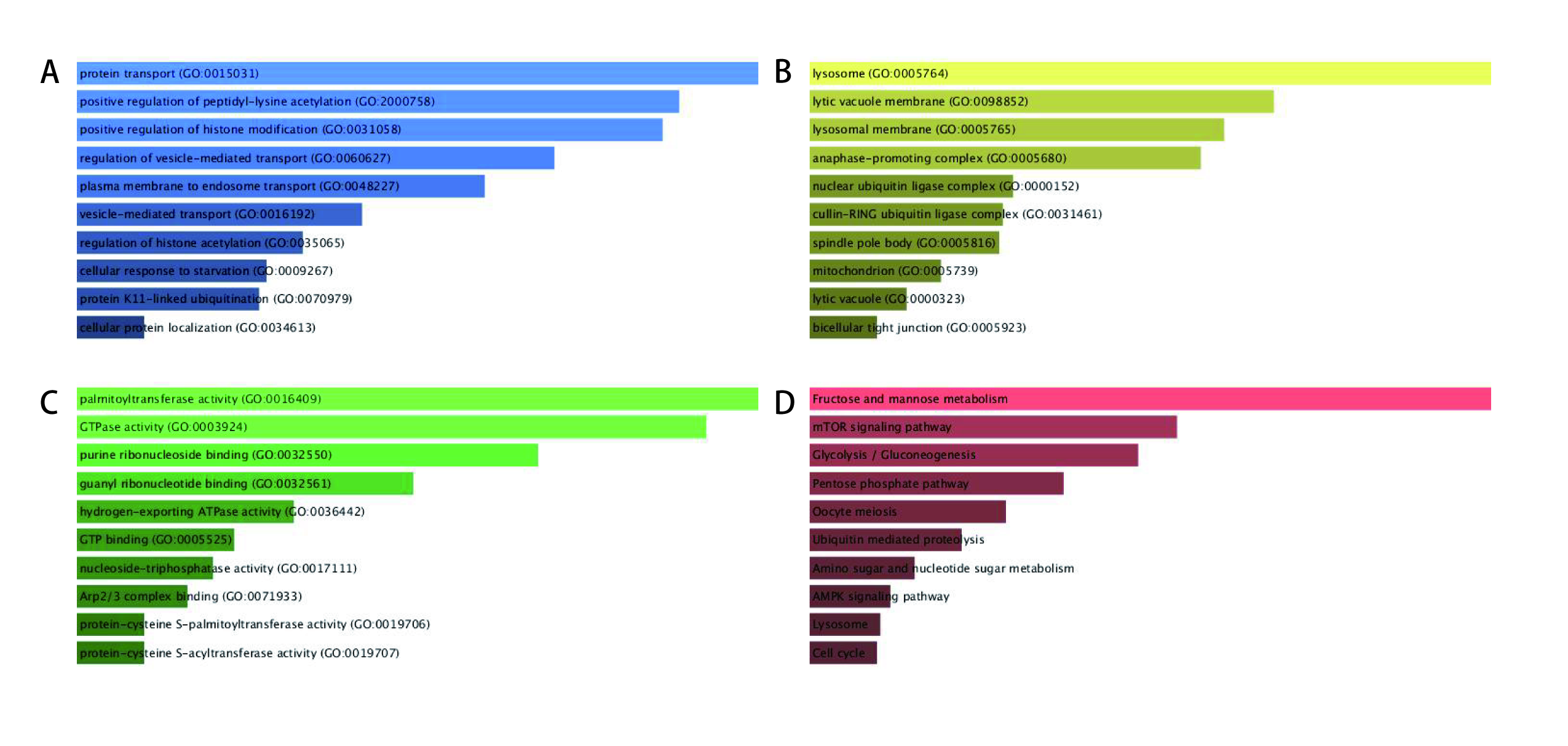

Supplement: Supplementary Figure 2 — GO and KEGG analysis (A) Top ten biological process related to key gene using GO analysis. (B) Top ten cellular component related to key genes using GO analysis. (C) Top ten molecular function related to key genes using GO analysis. (D) Top ten human pathway related to key genes using KEGG analysis. [file Image_2.tif]

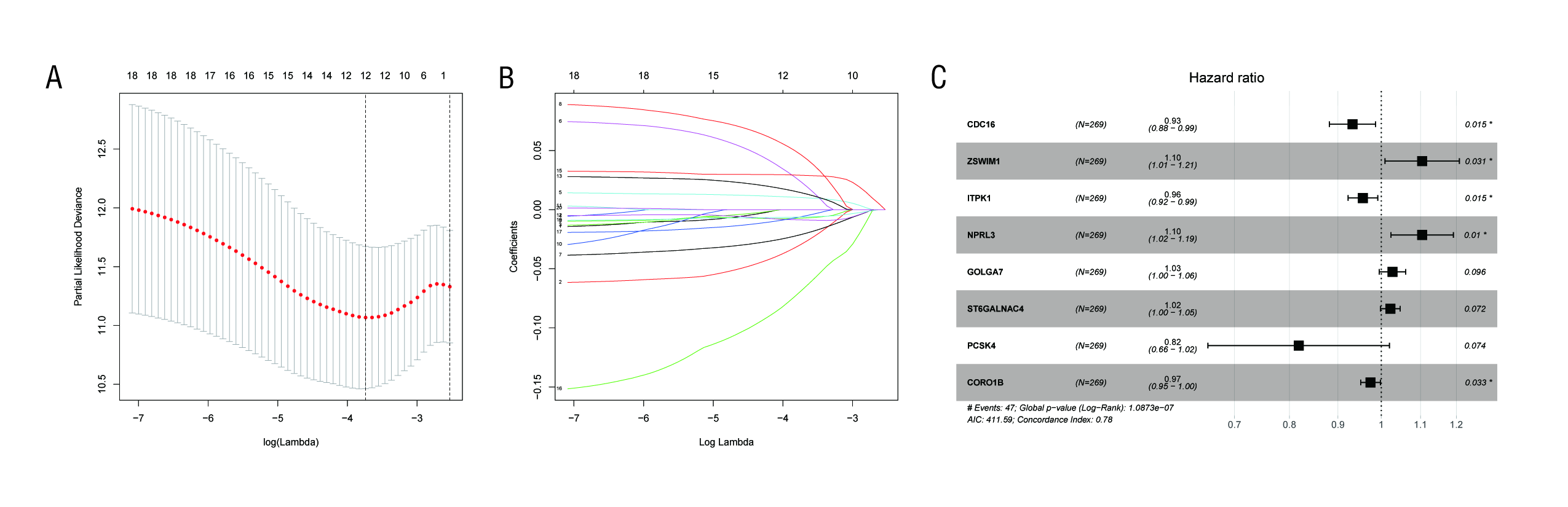

Supplement: Supplementary Figure 3 — LASSO COX regression and multivariate Cox analysis. (A, B) LASSO COX regression for OS of TRRS in the TCGA cohort. (C) Forest plot illustrating the multivariate Cox model results of eight gene related to Tregs. [file Image_3.tif]

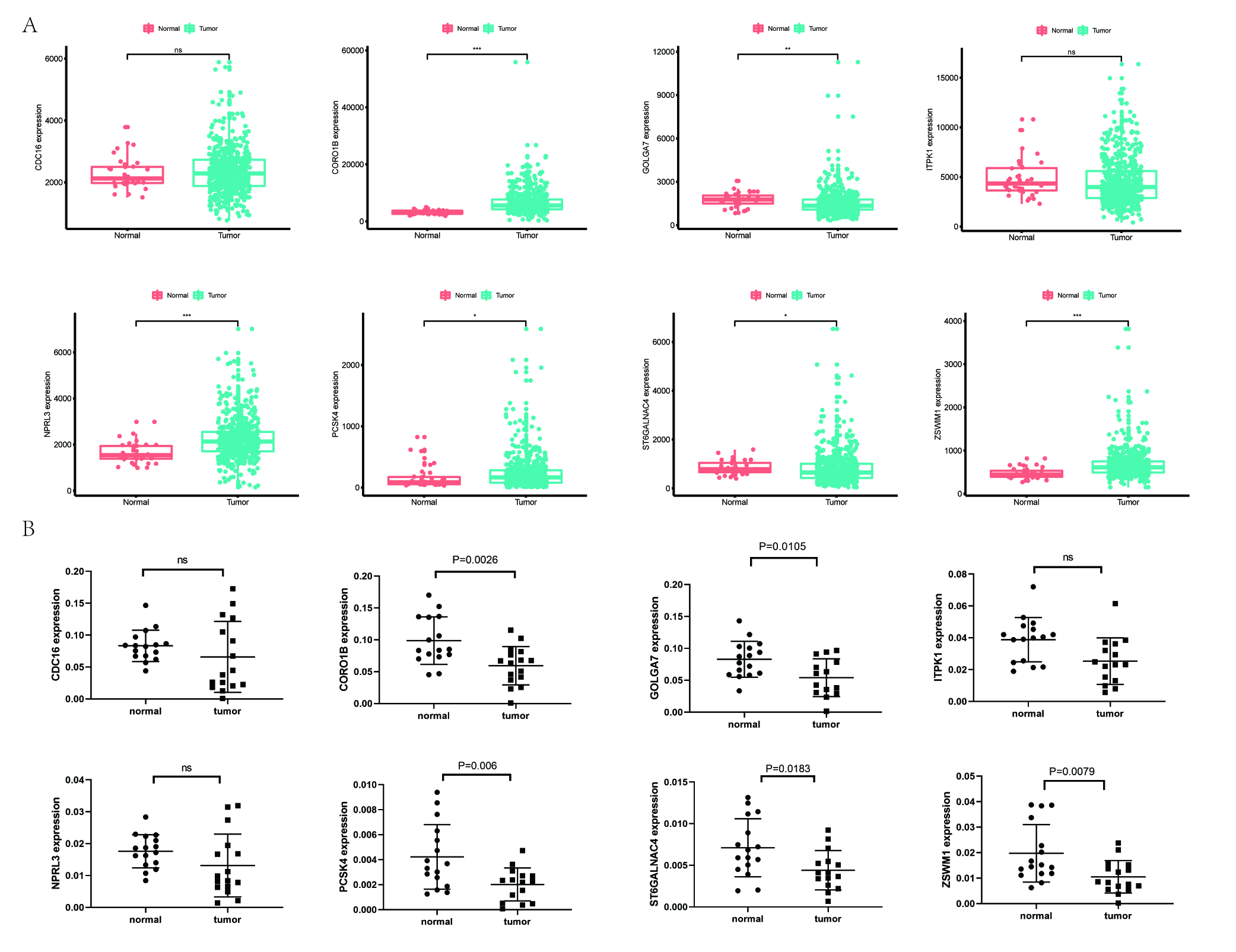

Supplement: Supplementary Figure 4 — Comparison of the expression of the eight genes. (A) Expression levels of the eight genes between EC samples and normal tissues evaluated by means of Wilcoxon signed-rank test. (B) Expression levels of the eight genes between normal tissues and tumor tissue evaluated by using qRT-PCR. [file Image_4.tif]

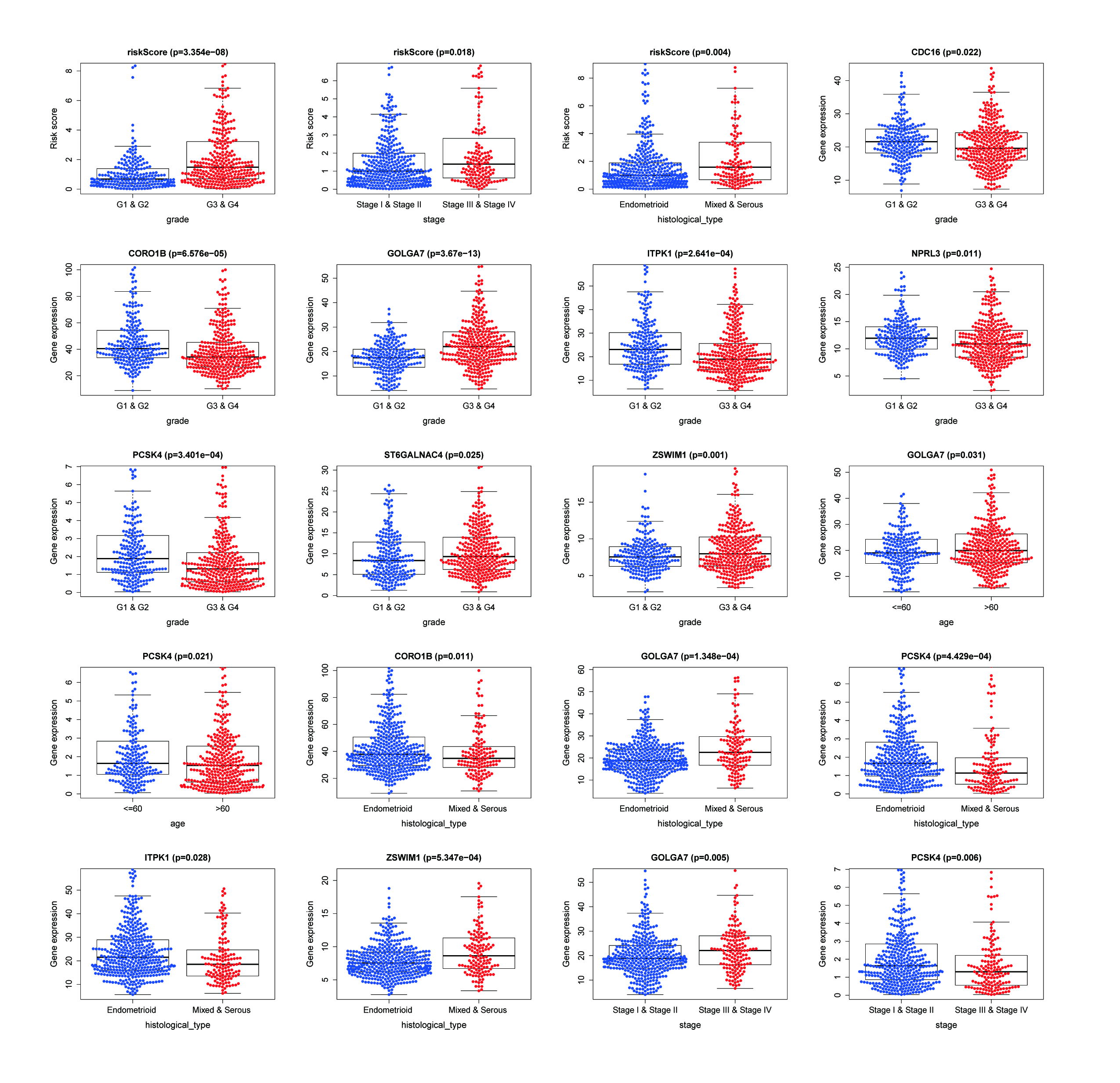

Supplement: Supplementary Figure 5 — The relationships between gene expression and clinical characteristic. [file Image_5.tif]

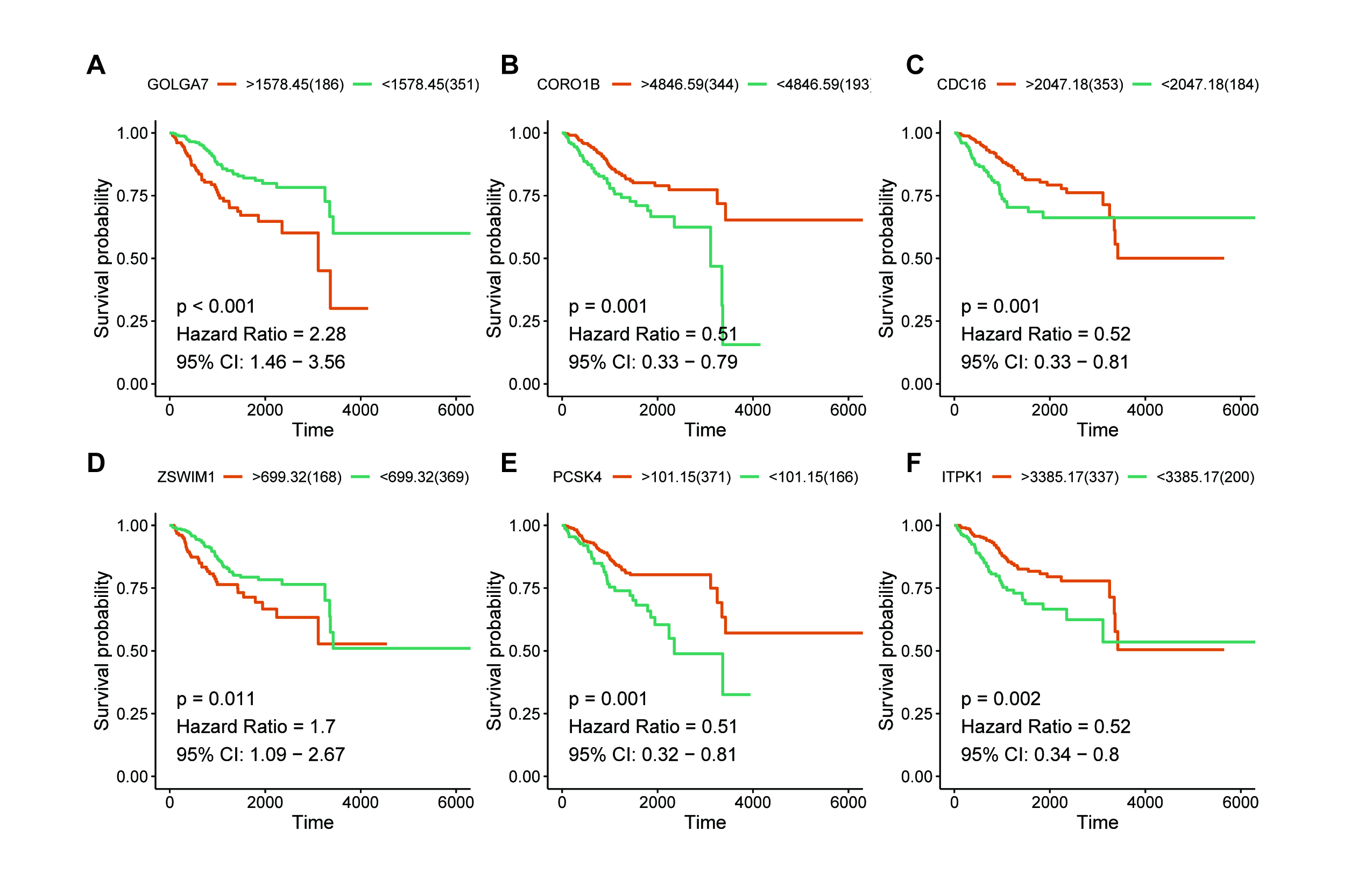

Supplement: Supplementary Figure 6 — The association of expression of eight gene and OS in TCGA UCEC dataset. (A) GOLGA7; (B) CORO1B; (C) CDC16; (D) ZSWIM1; (E)PCSK4; (F)ITPK1. [file Image_6.tif]

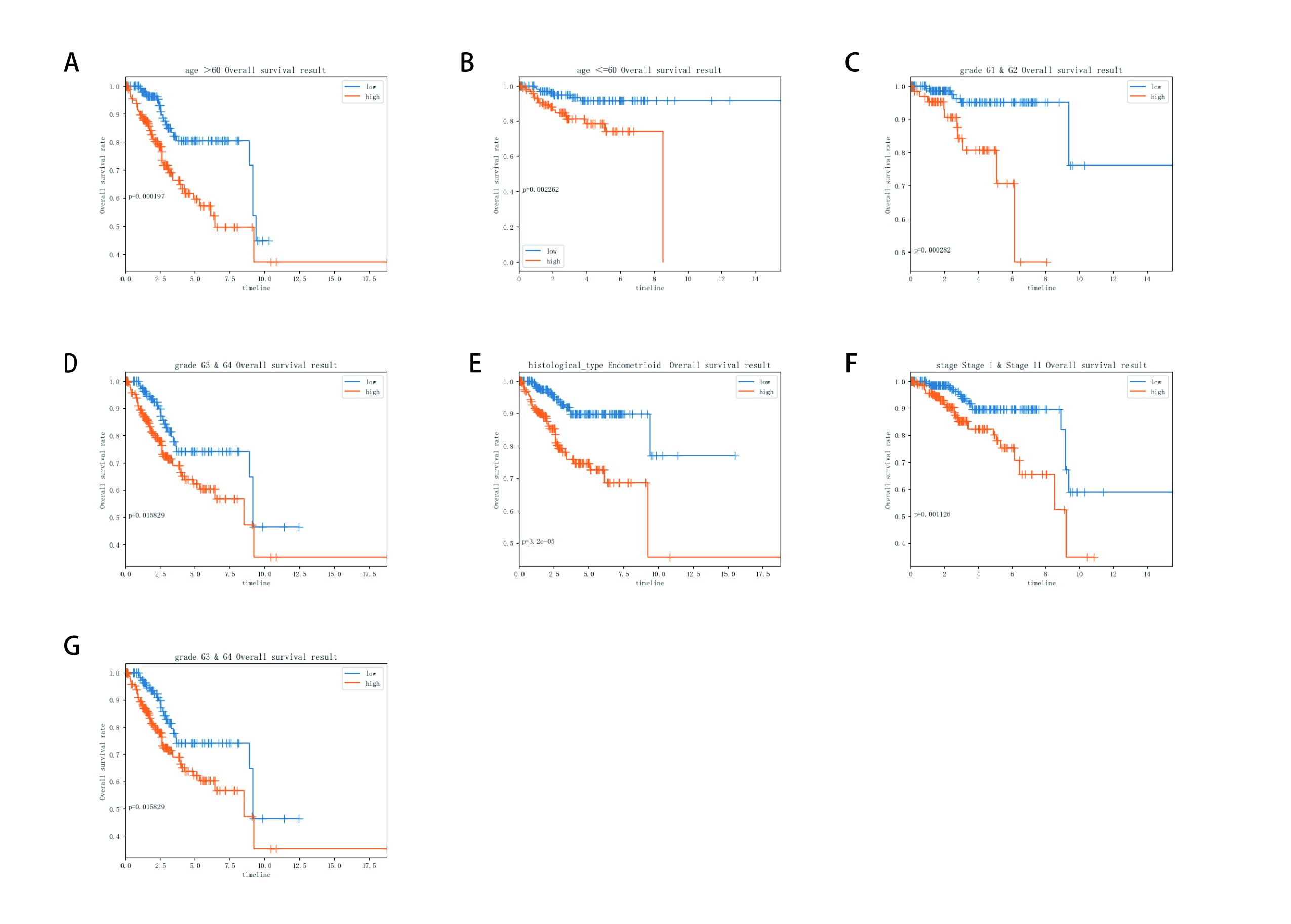

Supplement: Supplementary Figure 7 — Overall survival result between patients with high and low risk. (A) age>60; (B) age<60; (C) grade G1&G2; (D) grade G3&G4; (E)histological-type Mixed &Serous; (F) Stage I& Stage II; (G) Stage III& Stage IV. [file Image_7.tif]
