# Supplementary material for: Development and Clinical Validation of Novel 8-Gene Prognostic Signature Associated With the Proportion of Regulatory T Cells by Weighted Gene Co-Expression Network Analysis in Uterine Corpus Endometrial Carcinoma
Source: Front Immunol. 2021 Dec 14;12:788431. doi: 10.3389/fimmu.2021.788431 (PMC8712567; doi:10.3389/fimmu.2021.788431)
Supplement: Supplementary file 8 [file Table_1.docx]

Table S1. Primers used in PCR application

| Gene | Forward primers | Reverse primer |
| --- | --- | --- |
| CDC16 | TCAAAGTGCTCTATTTTGGGCA | TTGTCCAGTTTTCGTGACCGA |
| ZSWIM1 | GCTGAATGGGCTCCTGATTAAG | CCCTTGGGTTATAGGTCCGGT |
| ITPK1 | ACCGCTCCAAGTCCTATGAG | GGGAAAGTCAAGCCGTTCTTC |
| NPRL3 | GCTGTGTGAGAACAACGTCTA | AAGGAGAACTTGGCAAGAACG |
| ST6GALNAC4 | TGTGAGGAGATCGTGGTCTATG | CAAAGTAGTGGTAAGGCACTGAG |
| PCSK4 | TCCAAGCAGTGGTACATGAAC | CGGGTCGTAGTCATTGAAGTC |
| CORO1B | CCAGTGCTATGAGGACATTCG | CTTCGTCGTTGTGAGGACACC |
| GOLGA7 | GTGTTCATTCAGCGAGACTACA | CAACCTTCGAGATATGACTGGC |
