# Supplementary material for: Development and Clinical Validation of Novel 8-Gene Prognostic Signature Associated With the Proportion of Regulatory T Cells by Weighted Gene Co-Expression Network Analysis in Uterine Corpus Endometrial Carcinoma
Source: Front Immunol. 2021 Dec 14;12:788431. doi: 10.3389/fimmu.2021.788431 (PMC8712567; doi:10.3389/fimmu.2021.788431)
Supplement: Supplementary file 9 [file Table_2.docx]

Table S2. Univariate Cox regression analysis of 251 genes.

| id | HR | HR.95L | HR.95H | pvalue |
| --- | --- | --- | --- | --- |
| ST6GALNAC4 | 1.033823 | 1.013885 | 1.054153 | 0.000815 |
| GOLGA7 | 1.036362 | 1.010374 | 1.063018 | 0.005842 |
| CRELD2 | 0.94048 | 0.898957 | 0.983921 | 0.007732 |
| PCSK4 | 0.725631 | 0.570542 | 0.922876 | 0.008944 |
| GPX4 | 0.995569 | 0.992106 | 0.999044 | 0.012495 |
| SNX12 | 1.068729 | 1.014394 | 1.125974 | 0.012532 |
| SLC9A3R2 | 0.983858 | 0.971024 | 0.996862 | 0.015134 |
| ITPK1 | 0.961895 | 0.931994 | 0.992755 | 0.015896 |
| CORO1B | 0.97648 | 0.956633 | 0.996738 | 0.023096 |
| ECI1 | 0.980765 | 0.964329 | 0.997481 | 0.024291 |
| GPR108 | 0.951297 | 0.91055 | 0.993868 | 0.025394 |
| NPRL3 | 1.078208 | 1.0085 | 1.152735 | 0.027232 |
| CDC16 | 0.946189 | 0.900497 | 0.994199 | 0.028498 |
| TUBB4B | 0.998043 | 0.996263 | 0.999827 | 0.031520 |
| GDI1 | 1.017172 | 1.001419 | 1.033172 | 0.032518 |
| ZSWIM1 | 1.089282 | 1.006228 | 1.179192 | 0.034567 |
| TUBGCP6 | 0.914225 | 0.837474 | 0.998011 | 0.045019 |
| COASY | 0.967259 | 0.936062 | 0.999495 | 0.046575 |
| ZDHHC24 | 0.85421 | 0.731314 | 0.997759 | 0.046781 |
| FN3K | 0.939744 | 0.88371 | 0.999332 | 0.047560 |
